# Environmental Factors and Multiple Sclerosis Severity: A Descriptive Study

**DOI:** 10.3390/ijerph110606417

**Published:** 2014-06-19

**Authors:** Daniele Mandia, Ottavia E. Ferraro, Guido Nosari, Cristina Montomoli, Elisabetta Zardini, Roberto Bergamaschi

**Affiliations:** 1 Interdepartmental Research Center for Multiple Sclerosis (CRISM), C. Mondino National Neurological Institute, Pavia 27100, Italy; E-Mails: danielemandia@yahoo.com (D.M.); guido.nosari01@universitadipavia.it (G.N.); elisabetta.zardini@mondino.it (E.Z.); 2 Unit of Biostatistics and Clinical Epidemiology, Department of Public Health, Experimental and Forensic Medicine, University of Pavia, via Forlanini, 2, Pavia 27100, Italy; E-Mails: ottavia.ferraro@unipv.it (O.E.F.); cristina.montomoli@unipv.it (C.M.)

**Keywords:** multiple sclerosis, environment, disease severity, vitamin D, sunlight exposure

## Abstract

Growing evidence suggests that environmental factors play a key role in the onset of multiple sclerosis (MS). This study was conducted to examine whether environmental factors may also be associated with the evolution of the disease. We collected data on smoking habits, sunlight exposure and diet (particularly consumption of vitamin D-rich foods) from a sample of 131 MS patients. We also measured their serum vitamin D concentration. The clinical impact of MS was quantified using the Multiple Sclerosis Severity Score (MSSS); MS was considered “severe” in patients with MSSS ≥ 6, and “mild” in patients with MSSS ≤ 1. The results showed a strong association between serum vitamin D concentration and both sunlight exposure (26.4 ± 11.9 ng/mL *vs.* 16.5 ± 12.1 ng/mL, *p* = 0.0004) and a fish-rich diet (23.5 ± 12.1 ng/mL *vs.* 16.1 ± 12.4 ng/mL, *p* = 0.005). Patients reporting frequent sunlight exposure had a lower MSSS (2.6 ± 2.4 h *vs.* 4.6 ± 2.6 h, *p* < 0.001). The mild MS patients reported much more frequent sunlight exposure (75% mild MS *vs.* 25% severe MS *p* = 0.004, Chi square test). A higher serum vitamin D concentration determined a lower risk of developing severe MS, adjusted for sunlight exposure (OR = 0.92 for one unit increase in vitamin D, 95% CI: 0.86–0.97, *p* = 0.005). A stronger inverse association emerged between frequent sunlight exposure and the risk of severe MS (OR = 0.26, 95% CI: 0.09–0.71, *p* = 0.009). Our data show that an appropriate diet and adequate expose to sunlight are associated with less aggressive MS.

## 1. Introduction

Multiple sclerosis (MS), which is the most common cause of non-traumatic neurological disability in young adults, is a chronic inflammatory demyelinating disease of the central nervous system in which myelin auto-reactive T-lymphocytes drive an inflammatory process, leading to secondary macrophage recruitment and subsequent myelin destruction. MS is therefore considered an autoimmune disorder. The disease probably has a multifactorial genesis and, accordingly, no single trigger of the autoimmunity has been identified. The course of MS is characterised by an initial relapsing-remitting (RR) phase during which acute episodes (attacks) alternate with partial or complete remissions (periods of stability). All the neurological functions (motor, cerebellar, brainstem, sensory, sphincter, visual, mental) can potentially be impaired during attacks. The RR phase is generally followed by the secondary progressive (SP) phase, which is characterised by progressive impairment (with or without superimposed relapses) that often leads to irreversible disability [1,2]. Progressive accumulation of disability from the onset of the disease is observed in only a minority of patients, whose disease course is defined as primary progressive (PP) [3].

The global prevalence of MS shows a latitude gradient, being lower near the equator (<5/100,000), and higher in northern countries (>100/100,000 in North America, Canada and northern Europe) [4]. Nevertheless, there are a few exceptions, which can probably be explained on a genetic basis, e.g., a low prevalence of MS in the Norwegian Sami population [5] and a markedly increased frequency of the disease in Sardinia [6]. 

The risk of MS is higher in women than in men (sex ratio 2.5:1). This is probably due to sex hormone pattern differences that expose women to autoimmune responses [7]. 

Genetics also seem to play a role in the pathogenesis of MS, with monozygotic twins having an estimated risk of 17%, compared with 4% in dizygotic twins. Nevertheless, dizygotic twins are at greater risk than siblings (2%), an inconsistency that might be explained by the action of environmental or even intrauterine factors [8]. 

Thus, MS can be considered a multifactorial [9,10,11] disease in which environmental factors may be responsible for triggering the autoimmune response in genetically susceptible individuals.

It is possible that these factors, in addition to playing a role in the onset of MS, also influence its evolution. Several previous studies have already identified environmental factors probably involved in MS pathogenesis. To date, these factors have been studied separately, while a pooled analysis allowing evaluation of their possible interactions is lacking.

We briefly review some of the environmental factors that may have a role in MS and analyse their effects on disease evolution in a cohort of MS patients.

### 1.1. Cigarette Smoking

The effect of smoking on the immune system is well known; smokers (and ex-smokers) have higher levels of C-reactive protein, fibrinogen and inflammatory factors (e.g., IL-6), and they may also show a dysregulation of B-cell and T-cell homeostasis [12]. The nitric oxide (NO) contained in cigarettes seems to cause mitochondrial damage, leading to axonal degeneration and oligodendrocyte necrosis [13]. Raised NO levels in cerebrospinal fluid have been found during MS relapses and during disease progression [14]. Carbon monoxide, also contained in cigarettes, acts at a peripheral level, blocking tissue oxygenation and thus leading to demyelination [15]. Cigarette smoking per se, rather than the nicotine contained in tobacco, seems to be the factor responsible for oxidative damage in smokers; indeed, nicotine, acting on α7-cholinergic receptors present on immune cells, may exert an immunosuppressive (and thus protective) effect [16,17]. Finally, there is proof that smokers have a higher risk of developing MS and progress more rapidly to higher levels of disability [18].

### 1.2. Vitamin D

Vitamin D exists in nature as ergocalciferol (vitamin D2) and cholecalciferol (vitamin D3, the only form absorbed orally). Human skin is rich in 7-dehydrocholesterol, which, when the skin is exposed to sunlight, specifically to ultraviolet B (UVB) radiation, is transformed into cholecalciferol. In short, a hepatic hydroxylation step mediated by several P450 cytochromes, e.g., CYP27A1, CYP3A4, CYP2J3 and, most of all, CYP2R1, results in the formation of 25-OH-cholecalciferol [25-(OH)D3]. This is followed by a renal hydroxylation step, mediated by CYP27B1, which results in the formation of 1α,25-dihydroxyvitamin D3 (1α,25-(OH)_2_D_3_), the active metabolite [19]. Vitamin D is known to have a role in gene expression, binding to the intracellular vitamin D receptor (VDR), which in turn binds to the retinoid X receptor creating a heterodimer that recognises specific base sequences in DNA, namely vitamin D response elements (VDREs). This is how vitamin D influences protein transcription, not only in calcium and phosphate metabolism, but also in many other processes, e.g., lithocolic acid (LCA) inactivation (activated VDRs induce expression of CYP3A, which detoxifies LCA, an enteric carcinogen) [20], keratinocyte differentiation (promoted by calcium initially, and then by vitamin D) and innate and adaptive immunity regulation. VDREs can actually be found on several immune cells, e.g., macrophages, lymphocytes and dendritic cells. Vitamin D induces apoptosis in B cells (with decreasing Ig production) [21] and, *in vitro*, suppresses the T-cell response by inducing naive T-cells to mature into regulatory T cells (Tregs), which have an anti-inflammatory effect [22]. Furthermore, vitamin D induces interleukin (IL)-10 synthesis [23] and suppresses IFN-γ [24] and IL-2 [25]. These effects are known to be stronger in females than in males, possibly thanks to a synergistic action of 17β-estradiol and 1α,25-(OH)2D3 [26]. VDRs and 1α-hydroxylase can be found in the pancreas, muscle, ovary breast and brain; in the central nervous system they are located both in neurons and in glia, mainly in the hypothalamus and substantia nigra [27]. 

The scientific community currently agrees that 30–40 ng/mL of 1α,25-(OH)_2_D_3_ should guarantee bone health and prevent fractures [28], while a level > 24 ng/mL is sufficient to improve muscle performance, and thus reduce the risk of falls [29]. Most scientists consider levels < 20 ng/mL to constitute a *deficiency,* and levels of 21–29 ng/mL to constitute *insufficiency* [30]. Moreover, levels < 15 ng/mL are associated with a higher risk of cardiovascular diseases [28]. An intake of 2.5 μg is needed to increase vitamin D blood levels by 1 ng/mL; we therefore need at least 75 μg/day in order to reach the safety threshold of 30 ng/mL [31]. Diet actually accounts for only a small part of our vitamin D supply: In fact, dietary intake in North America and Europe is currently 2.5–5 μg/day, whereas the European recommendations advocate suggest 20 μg/day [32]; most of our vitamin D is made in the skin through exposure to sunlight (specifically UVB radiation) [33]. Thus, the risk of vitamin D deficiency is higher in subjects who have little or no exposure to sunlight, e.g., people who work indoors, the elderly [34], people who live beyond the 40th parallel [35]. However, obesity also confers a higher risk: Adipocytes, in fact, capture circulating vitamin D, decreasing its serum concentration [36]. 

An important long-term study of 7 million US military personnel showed a negative association between high levels of 1α,25-(OH)_2_D_3_ and risk of MS [37]. A retrospective Italian study of subjects with a first clinical event suggestive of MS found that those with low serum vitamin D had a higher risk of developing clinically definite MS [38]. Moreover, vitamin D insufficiency also seems to have a role in promoting long-term MS activity and progression [39].

### 1.3. Sunlight Exposure

A recent meta-analysis has demonstrated an inverse association between MS risk and exposure to UV radiation (at least 20-fold stronger than the association between MS risk and other environmental factors) [40]. UV radiation can help to reduce MS risk not only by producing vitamin D, but also through other pathways; indeed, it probably induces IL-10, TNF-α and Treg cells, all of which can ultimately have anti-inflammatory effects [41]. However, MS cannot be explained by lack of sunlight exposure alone. Indeed, an increased prevalence of MS in spite of adequate UV radiation exposure has been found in some Italian regions, where it may be due to a high frequency of HLA-DRB1, an allele associated with a higher MS risk, which may therefore constitute a genetic predisposition factor. An unusual relationship has also been found in northern Scandinavia, *i.e.*, a lower MS prevalence in spite of the weak sunlight. This finding can be explained by the fact that this population has a much higher dietary vitamin D intake than that found in the rest of Europe (6–9.9 μg/day *vs.* 2–3.3 μg/day) [42], and it further strengthens the hypothesis that MS has a multifactorial pathogenesis. 

### 1.4. Diet

Although many studies have sought to identify a dietary regimen that may lower the risk of MS or MS relapses [43,44,45], no conclusive evidence has yet been produced. The only prospective study conducted to date monitored dietary vitamin D intake in a cohort of more than 180,000 people for a period of 20 years. It showed that high vitamin D levels may have a protective effect against the risk of developing MS [46].

## 2. Patients and Methods

### 2.1. Patients

Our cohort comprised 131 subjects diagnosed with MS according to the 2001 McDonald criteria [47] and consecutively seen at our MS centre (Table 1) in the period January 2013–January 2014.

Each patient, having first signed the informed consent form, underwent a neurological examination and the administration of three questionnaires; a blood sample was also taken for 25-(OH)D_3_ measurement (1α,25-(OH)_2_D_3 _ was not measured because of its extremely short half-life) [48].

Neurological disability was quantified using the Expanded Disability Status Scale (EDSS) [49]. This is a widely used and validated scale that synthetically expresses the level of disability deriving from the involvement of several neurological systems (motor, cerebellar, brainstem, sensory, bowel and bladder, visual, mental). It ranges from 0 (no neurological abnormality) to 10 points (death from MS). Values over 4.0 are associated with impaired gait.

To assess the clinical impact of MS, we used the Multiple Sclerosis Severity Score (MSSS), which describes the severity of the disease at any given time. The MSSS is calculated using an algorithm that adjusts the EDSS score for disease duration [50].

The characteristics of the MS patients are reported in Table 1.

**Table 1:** Demographic and Clinical Features of 131 MS Patients.

Sex	81 Women/50 Men
Age	Mean 45.2 ± SD 11.0 (range 17–73)
Disease duration	Mean 13.6 ± SD 9.1 (range 1–42)
EDSS score	Mean 3.0 ± SD 2.4 (range 0–8)
MSSS	Mean 3.2 ± SD 2.6 (range 0.05–9.5)

(EDSS = expanded disability status scale; MSSS = multiple sclerosis severity scale).

### 2.2. Data Collection

Environmental factors were assessed using three questionnaires investigating: (1) lifetime smoking habits, (2) diet, particularly consumption of vitamin D-rich foods (e.g., beef liver, milk, eggs, cheese, *etc.*) in the previous two years and vitamin D supplementation, (3) sunlight exposure in the previous two years. Table 2 summarises the information gathered through these questionnaires.

**Table 2:** Information Collected through Questionnaires.

Variables	Negative	Positive
Vegetarian	No	Yes
Egg consumption	No/rarely	> 3–6 times/month
Fish consumption	No/rarely	> 3–6 times /month
Consumption of dairy products	No/rarely	> 3–6 times /month
Liver consumption	No/rarely	> 3–6 times /month
Vitamin supplementation^1^	No	Yes
Consumption of fortified foods	No	Yes
Sunlight exposure during the year	None/rare	Frequent^2^
Sunlight exposure per day	<2 h/day	>2 h/day
Sunscreen use	No	Yes
Ever smoking	No	Yes
Current smoking	No	Yes
Passive smoking (previous 12 months)	No	Yes

^1^ Given the variety of vitamin D dosages, these are not indicated; we merely identified patients taking cholecalciferol as a vitamin supplement. ^2^ Frequent sunlight exposure corresponds to exposure for more than one h/day at weekends and during holidays

In addition, a blood sample (3–4 mL) was taken from each patient to measure the 25(OH)-D concentration in ng/mL. Because of its short half-life, 1α,25-(OH)_2_D_3_ was not measured.

### 2.3. Statistical Analysis

Variables were expressed using means and standard deviations. To compare smoking and dietary habits between different classes of disease severity (mild *vs.* severe patients), an unpaired t test for quantitative variables and chi-squared test or Fisher’s exact test for qualitative ones were used. Odds ratios (OR) and corresponding 95% confidence intervals (95% CI) were used to analyse the association between environmental factors and MS severity. A multiple linear regression model containing demographic and environmental variables was performed to identify relevant predictors correlated with MS severity, adjusting for age and sex. A *p*-value less than 0.05 was considered significant (two-sided). All the analyses were performed using STATA^®^ 12 software (Stata Corporation, 4905 Lakeway Drive, College Station, TX 77845-4512, USA, 2012).

## 3. Results

No significant differences in MS severity were found between smokers and ex- or never-smokers. Furthermore, no significant relationship between passive smoking and MSSS was found (Table 3).

**Table 3:** Smoking habits and MS severity (MSSS mean value ± standard deviation).

VARIABLES	MSSS MEAN ± SD	*p*-value
**Ever smoking**		
Yes	3.2 ± 2.6	n.s.
No	3.0 ± 2.5	
**Smoking status**		
Never-smoker	3.0 ± 2.5	n.s.
Ex-smoker	3.7 ± 2.6	
Smoker	2.5 ± 2.5	
**Smoking exposure (active/passive)**		
Yes	2.8 ± 2.4	n.s.
No	3.4 ± 2.7	

No associations emerged between diet and severity of MS, with the sole exception of liver consumption; indeed, frequent liver consumption was related to more severe disease (*p* = 0.03); however, it has to be considered that only eight subjects claimed to eat liver (Table 4).

**Table 4:** Diet and MS Severity (MSSS value).

Variables	MSSS Mean ± SD	*p*-value
**Vegetarian**		
Yes (2)	3.2 ± 2.6	n.s.
No (130)	3.2 ± 2.6	
**Egg consumption**		
Yes (102)	3.4 ± 2.7	n.s.
No (30)	2.8 ± 2.3	
**Fish consumption**		
Yes (104)	3.2 ± 2.6	n.s.
No (28)	3.3 ± 2.8	
**Consumption of dairy products**		
Yes (114)	3.1 ± 2.6	n.s.
No (18)	4.1 ± 2.5	
**Liver consumption**		
Yes (7)	5.1 ± 2.5	0.03
No (125)	3.1 ± 2.6	
**Vitamin supplementation**		
Yes (61)	3.2 ± 2.6	n.s.
No (70)	3.3 ± 2.6	
**Fortified foods**		
Yes (30)	2.9 ± 2.5	n.s.
No (101)	3.4 ± 2.63	

The mean MSSS was significantly lower in subjects who reported frequent sunlight exposure as opposed no or rare exposure (2.6 *vs.* 4.6; *p* < 0.001); correspondingly, lower MSSS values were found in subjects who had more, as opposed to less, than two hours’ sunlight exposure per day (2.8 *vs.* 3.9; *p* = 0.02). Sunscreen use was significantly correlated with increased sunlight exposure (*p* = 0.008), but no association was found with MSSS (Table 5).

**Table 5:** Sunlight Exposure and MS Severity (MSSS Value).

VARIABLES	MSSS MEAN ± SD	*p*-value
**Sunlight exposure**		
Frequent	2.6 ± 2.4	<0.001
None/rare	4.6 ± 2.6	
**Hours/day of sunlight exposure**		
>2 h/day	2.8 ± 2.4	0.02
<2 h/day	3.9 ± 2.7	
**Sunscreen use**		
Yes	2.9 ± 2.3	n.s.
No	3.7 ± 2.8	

Serum vitamin D concentrations were higher in subjects who had a diet rich in fish (mean 23.5 ng/mL *vs.* 16.1 ng/mL; *p* = 0.005). No correlation was found between vitamin D concentration and any other dietary component (e.g., dairy products, liver, eggs, vitamin D supplements, fortified foods).

Subjects who reported frequent sunlight exposure had higher serum vitamin D levels (24.6 ng/mL *vs.* 16.5 ng/mL; *p* = 0.0004); a similar result was found in subjects having more than two hours’ sunlight exposure per day (24.7 ng/mL *vs.* 18.3 ng/mL; *p* = 0.004) (Table 6). No association was found between smoking habits and vitamin D levels.

**Table 6:** Vitamin D levels (expressed in ng/mL) according to smoking habit, diet, sun exposure.

VARIABLES	MEAN (ng/mL) ± SD	*p*-value
**Vegetarian**		
Yes	20.8 ± 14.1	n.s.
No	21.9 ± 12.5	
**Egg consumption**		
Yes	22.1 ± 12.3	n.s.
No	21.2 ± 13.1	
**Fish consumption**		
Yes	23.5 ± 12.1	0.005
No	16.1 ± 12.4	
**Consumption of dairy products**		
Yes	21.9 ± 13.0	n.s.
No	21.6 ± 9.4	
**Liver consumption**		
Yes	24.7 ± 16.7	n.s.
No	21.7 ± 12.2	
**Vitamin supplementation**		
Yes	22.5 ± 11.9	n.s.
No	20.9 ± 12.6	
**Consumption of fortified foods**		
Yes	20.9 ± 11.1	n.s.
No	22.1 ± 12.7	
**Ever smoking**		
Yes	21.2 ± 11.6	n.s.
No	23.5 ± 13.1	
**Smoking status**		
Never-smoker	23.5 ± 13.1	n.s.
Ex-smoker	20.3 ± 12.0	
Smoker	22.5 ± 11.0	
**Smoking exposure (active/passive)**		
Yes	21.0 ± 11.2	n.s.
No	22.3 ± 13.1	
**Sun exposure**		
Frequent	24.6 ± 11.9	0.0004
None/rare	16.5 ± 12.1	
**Hours/day of sunlight exposure**		
>2 h/day	24.7 ± 11.5	0.004
<2 h/day	18.3 ± 13.0	
**Sunscreen use**		
Yes	24.2 ± 11.9	0.02
No	19.2 ± 12.7	

Thirty-six patients with mild MS (MSSS ≤ 1) were compared with 30 patients with severe MS (MSSS ≥ 6) (Table 7).

**Table 7:** Comparison of patients with mild *vs.* severe MS, according to smoking habits, diet, sunlight exposure.

Variables	MSSS ≤ 1	MSSS ≥ 6	*p*-value
**Vitamin D**			
Mean (SD)	22.8 (10.6)	12.4 (10.7)	0.0002
**Egg consumption**			
Yes	25 (69.4%)	26 (86.7%)	n.s.
No	11 (30.6%)	4 (13.3%)	
**Fish consumption**			
Yes	29 (80.6%)	23 (76.7%)	n.s.
No	7 (19.4%)	7 (23.3%)	
**Consumption of dairy products**			
Yes	32 (88.9%)	25 (83.3%)	n.s.
No	4 (11.1.%)	5 (16.7%)	
**Liver consumption**			
Yes	1 (2.8%)	4 (13.3%)	n.s.
No	35 (97.2%)	26 (86.7%)	
**Vitamin supplementation**			
Yes	19 (52.8%)	12 (40%)	n.s.
No	17 (47.2%)	18 (60%)	
**Fortified foods**			
Yes	11 (30.6%)	5 (17.2%)	n.s.
No	25 (69.4%)	24 (82.8%)	
**Ever smoking**			
Yes	21 (58.3%)	18 (69.2%)	n.s.
No	15 (41.7%)	8 (30.8%)	
**Smoking status**			
Never-smoker	15 (41.7%)	8 (30.8%)	n.s.
Ex-smoker	11 (30.5%)	12 (46.1%)	
Smoker	10 (27.8%)	6 (23.1%)	
**Passive smoking**			
Yes	12 (33.3%)	9 (31.0%)	n.s.
No	24 (66.7%)	20 (69.0%)	
**Sunlight exposure**			
Frequent	27 (75%)	12 (40%)	0.004
None/rare	9 (25%)	18 (60%)	
**Hours of sunlight exposure per day**			
>2 h/day	25 (69.4%)	11 (36.7%)	0.008
<2 h/day	11 (30.6%)	19 (63.3%)	
**Sunscreen use**			
Yes	22 (61.1%)	11 (36.7%)	0.048
No	14 (38.9%)	19 (63.3%)	

Vitamin D concentration (22.8 ng/mL *vs.* 12.4 ng/mL) and sunlight exposure were significantly higher in the mild MS patients; the risk of having severe MS, defined as MSSS ≥ 6, was about 75% less in the subjects who received more than two h/day of sunlight compared with those reporting less than two h/day (OR = 0.26, 95% CI: 0.09–0.71, *p* = 0.009).

As regards the impact of environmental factors on the risk of having severe MS, the most important finding was that vitamin D concentration had an OR of 0.92 (95% CI: 0.86–0.97, *p* = 0.005), adjusted for sunlight exposure: this means that a 5 ng/mL increase in vitamin D concentration may correspond to a 34% reduction in the probability of having severe MS (0.92^5^ = 0.66).

A multiple linear regression model containing demographic (age and sex) and environmental (vitamin D levels, sun exposure, smoking, diet) variables was fitted. Table 8 reports the final model in which only significant independent variables and confounders are shown. MS severity was inversely correlated with both vitamin D intake and sunlight exposure, after controlling for age and sex.

**Table 8:** Regression coefficients for developing “Severe” MS, according to environmental factors.

Variables	Regression Coefficient	Standard Error	95% CI		*p*-value
Gender	−0.05	0.41	−0.85	0.75	0.901
Age	0.07	0.02	0.03	0.10	<0.001
Vitamin D	−0.06	0.02	−0.09	0.02	0.001
Sun exposure	−1.26	0.44	−2.13	−0.39	0.005

## 4. Discussion

The purpose of this study was to investigate environmental factors in MS, focusing not on the absolute risk of MS (already evaluated in several previous studies) [39,51,52,53], but on the severity of the disease. Some trials investigating the relationship between risk factors and MS severity simply considered MS type (RR, SP, PP) or disability as quantified by the EDSS [54]. Other studies instead evaluated only the association between vitamin D and risk of relapse in RR MS patients [55,56]. In our view, however, the clinical impact of MS should be evaluated taking into account not only the neurological disability caused by the disease but also the period of time over which this disability develops. It has to be appreciated that the same level of neurological disability can be seen both in aggressive forms of MS, in which it develops within months of the clinical onset, and in relatively mild forms, in which it develops after decades of the disease. For this reason, the disability needs to be adjusted for disease duration. However, this is not straightforward. One proposed solution was to calculate a so-called progression index (the ratio of EDSS score to time from onset), but this approach was complicated by the fact that the EDSS is not a linear measure of disability. The MSSS corrects the EDSS for disease duration using an arithmetically simple method, which compares individual disability with the distribution of scores in cases having equivalent disease duration [50]. In this study, we therefore used the MSSS to detect different rates of disease progression, considering it more powerful than other methods. The other novel aspect of our work is the fact that, unlike previous studies, we investigated several environmental factors not separately but in the same cohort of MS patients.

The risk of developing MS is known to be greater at higher latitudes, where the sunlight is weaker; a greater risk of MS has also been reported in subjects with lower vitamin D levels [35]. Furthermore, most MS patients have been found to have a vitamin D deficiency regardless of their MS type [54]. A prospective study performed in the USA in a cohort of 200,000 women showed that, over a period of 20 years, the incidence of MS was inversely associated with vitamin D intake [46]. A recent Italian study has shown, on the other hand, that subjects at onset of MS have lower vitamin D levels, and the same has been found in patients with frequent relapses [38].

We therefore studied the influence of vitamin D considering: its serum concentration and its intake both through diet and through sunlight exposure. Our results show that vitamin D plays a role in the evolution of MS: an inverse relationship was found between lower vitamin D concentrations and disease severity (as assessed by MSSS). A fish-rich diet was found to be significantly associated with higher vitamin D levels: in fact, fish is among the foods with a higher cholecalciferol content. In northern Scandinavia, higher dietary vitamin D intake (through a diet rich in bluefish) compensates for the lack of sunlight. Conversely, we found that none of the other vitamin D-rich foods (dairy products, liver, eggs) exerted a significant influence. However, it must be remembered that 80% of cholecalciferol is synthesised in the skin, thanks to UVB radiation. Our results seem to confirm this: the subjects who had more than two hours’ sunlight exposure per day were less likely to develop a severe form of MS. Although sunscreen serves to protect against UV radiation, we found sunscreen use to be associated with a better disease prognosis. This is probably because sunscreen use is an expression of prolonged sunlight exposure. Accurate assessment of sunlight exposure is actually very difficult, given that the patient’s subjective evaluation of it may not be reliable. This is a bias that only a prospective study might potentially be able to overcome, even though such a study would be difficult to perform.

Nevertheless, we observed that an increased vitamin D concentration decreased the risk of having a severe form of MS. Vitamin D is in fact known to be a modulator of the immune system, as it stimulates Tregs and suppresses inflammatory cytokines, e.g., IL-17 and IFN-γ [57].

A weak point of the present study, and of other studies that have detected an inverse relationship between MS severity and both sunlight exposure and vitamin D levels, is that it cannot be established whether high vitamin D levels are a cause or an effect of mild MS: indeed, patients with less disability are likely to spend more time outdoors, in the sunlight, whereas patients with a severe form, and therefore more disability, probably spend more time indoors. Nevertheless, prospective studies [39] indicated that vitamin D levels are able to predict long-term outcome of MS. Anyway, the question of whether vitamin D has a causal role or is just a marker for some other factor which influences MS severity is still open, and it is one that only ongoing randomised clinical trials might answer.

As for cigarette smoking, several trials have already shown that it is strongly related to more severe forms of MS, and might promote evolution of the disease to a progressive form [58]. On the other hand, recent research has shown a “protective” role of nicotine [17]. In our research, rather than simply considering smokers *vs.* non-smokers, we quantified the patients’ cigarette addiction and also considered the role of passive smoking. Despite this, we did not find any association between smoking and MS severity; we did not have data allowing us to evaluate tobacco snuff use, which seems to exert a “protective” role.

Considering the complex involvement of different factors in determining the clinical evolution of MS, sunlight exposure, diet and vitamin D alone are, singly, unlikely to determine the outcome of MS. They are more likely cofactors, acting together with other environmental and non-environmental factors (e.g., genetics).

## 5. Conclusions

The results of our study indicate that adequate vitamin D levels and frequent exposure to sunlight are associated with relatively mild MS, and therefore suggest that these environmental factors could play a role in the pathogenesis of this disease. There are also other factors (e.g., pollution, organic solvents) that could exert a negative influence. 

The relatively small sample size could be a limiting factor of the present study. Prospective evaluations better designed to consider the role of environmental factors might, in the future, help to further improve our understanding of the pathogenesis of MS, and open the way for the development of new models of prevention and therapy. 
